# Variability of Isavuconazole Trough Concentrations during Longitudinal Therapeutic Drug Monitoring

**DOI:** 10.3390/jcm11195756

**Published:** 2022-09-28

**Authors:** Léa Bolcato, Anne Thiebaut-Bertrand, Françoise Stanke-Labesque, Elodie Gautier-Veyret

**Affiliations:** 1 Laboratory of Pharmacology, Pharmacogenetics and Toxicology, Grenoble Alpes University Hospital, 38000 Grenoble, France; 2 Clinical Hematology Department, Grenoble Alpes University Hospital, 38000 Grenoble, France; 3 Faculty of Medicine, University Grenoble Alpes, Inserm, U1300, CHU Grenoble Alpes, 38000 Grenoble, France

**Keywords:** isavuconazole, therapeutic drug monitoring, trough concentration, pharmacokinetics

## Abstract

Isavuconazole (ISA), a triazole antifungal agent, is licensed for the treatment of invasive aspergillosis and mucormycosis. Therapeutic drug monitoring (TDM) is a cornerstone of treatment efficacy for triazole antifungals due to their pharmacokinetic variability, except for ISA, for which the utility of TDM is still uncertain. We performed a retrospective study that aimed to assess the inter- and intra-individual variability of ISA trough concentrations (Cmin) and to identify the determinants involved in such variability. ISA Cmin measured in adult patients at the Grenoble Alpes University Hospital between January 2018 and August 2020 were retrospectively analyzed. In total, 304 ISA Cmin for 33 patients were analyzed. The median ISA Cmin was 2.8 [25th–75th percentiles: 2.0–3.7] mg/L. The inter- and intra-individual variability was 41.5% and 30.7%, respectively. Multivariate analysis showed independent covariate effects of dose (β = 0.004 ± 3.56 × 10^−4^, *p* < 0.001), Aspartate aminotransférase (ASAT) (β = 0.002 ± 5.41 × 10^−4^, *p* = 0.002), and protein levels (β = 0.022 ± 0.004, *p* < 0.001) on ISA Cmin, whereas C reactive protein levels did not show any association. This study, conducted on a large number of ISA Cmin, shows that ISA exposure exhibits variability, explained in part by the ISA dose, and ASAT and protein levels.

## 1. Introduction

Isavuconazole (ISA) is a broad-spectrum triazole antifungal agent licensed in adult patients for the treatment of invasive aspergillosis and mucormycosis [1]. This antifungal agent is available by both intravenous and oral administration, with excellent bioavailability (almost 98%) [2,3]. ISA exhibits dose-proportional pharmacokinetics characterized by slow metabolism by cytochrome P450 (CYP) 3A4 and 3A5, resulting in a long elimination half-life (80–120 h) [4]. ISA has a very high affinity (>99%) for human plasma proteins, including albumin. In light of the high pharmacokinetic variability of triazole antifungals, therapeutic drug monitoring (TDM) is a cornerstone of antifungal treatment, especially for voriconazole [5,6]. However, because of its particular pharmacokinetic properties, TDM for ISA is currently only recommended in cases of therapeutic inefficacy, adverse effects, or drug-drug interactions [5]. Indeed, phase III studies did not shown any link between ISA exposure and efficacy or toxicity, with ISA trough concentrations (Cmin) being less variable than those observed for other antifungal triazoles [7]. Conversely, real-life studies have reported variable ISA exposure (coefficients of variation of 51 and 59% for area under the curve and Cmin of ISA at inter-individual level) [8] and a variable relationship between ISA Cmin and side effects [9,10].

These differences between the phase III and real-life studies [7,9,10,11] could be explained by different study populations, with more patients with comorbidities in real life. In terms of the factors involved in such variability, older age [10], larger dose [10], low body-mass index [8], liver failure [9,12], Asian race [12], and even a longer duration of treatment [9], have been shown to be associated with increased ISA exposure. Conversely, female gender, hemodialysis, or comedication with CYP3A4 inducer, appear to be associated with low ISA exposure [8,13]. Further studies on larger numbers of patients are needed to clarify the variability of ISA exposure and the possible associated factors. Moreover, the impact of the inflammatory status on ISA Cmin has not yet been studied, although it is now clearly accepted to be a major determinant of voriconazole Cmin through the inhibitory effect of inflammation on CYP transcription and activity [14,15,16,17].

The objective of this study was to therefore evaluate the inter- and intra-individual variability of ISA Cmin and identify determinants involved in such variability, including for the first time the impact of inflammation on ISA Cmin.

## 2. Materials and Methods

### 2.1. Study Design and Data Collection

This monocentric retrospective study was conducted at the Grenoble Alpes Medical Center from January 2018 to August 2020, and was approved by the Grenoble University Hospital review board (registration RnIPH 2020, protocol ISAVAR; CNIL number: 2205066 v0). Study ethics approval was obtained on 7 September 2021 (CECIC Rhône-Alpes-Auvergne, Clermont-Ferrand, IRB 5891). All adult patients (>18 years of age) followed at the Grenoble Alpes University Hospital and who were treated with ISA with at least one ISA Cmin determination during the period of interest, were eligible. Patients were excluded in cases of prescription error, concomitant treatment with a strong enzyme inducer or inhibitor, and if treatment had been discontinued or a pharmacokinetic steady state had not been achieved at the moment of ISA determination (at least three days after the initiation of therapy with a complete loading dose, or after 7 days after a dose adjustment or change of route of administration).

Demographic (age, sex, and weight), clinical (underlying disease and indication of ISA), biological (ISA Cmin, C-reactive protein (CRP), aspartate aminotransferase (ASAT), alanine aminotransferase (ALAT), gamma glutamyltransferase (GGT), alkaline phosphatase (ALP), total bilirubin, creatinine, total protein, albumin, lactate dehydrogenase (LDH) levels, and pharmaceutical (date of initiation of ISA treatment, route of administration, maintenance daily dose, and treatment duration) data were retrospectively collected from medical records.

### 2.2. Therapeutic Drug Monitoring of ISA

ISA TDM was performed on samples taken at least 20 h after the last drug dose. ISA plasma Cmin were determined using a validated liquid chromatography-tandem mass spectrometry method adapted from our previously published method [18]. This method allows ISA quantification in plasma from 0.1 to 20 mg/L, with good precision (intra- and inter-day coefficient of variation <15%) and accuracy (intra and inter-day biases ± 15%). ISA Cmin are considered therapeutic in our institution if they are between 2 and 5 mg/L [9,19] and any dose adjustment was left to the discretion of the clinician.

### 2.3. Treatment Response and Adverse Effects

The treatment response was assessed three months after the initiation of ISA treatment according to the 2008 Mycoses Study Group and European Organization for Research and Treatment of Cancer consensus criteria, only in patients with hematological malignancies curatively treated [20]. Patients were classified into three groups according to the evolution of their clinical and radiological signs: complete or partial therapeutic response or failure. A complete response is defined as the full resolution of clinical and radiological signs. A partial therapeutic response is defined as a major improvement in clinical signs associated with an at least 25% reduction in the diameter of radiological lesions. Treatment failure includes stable response (survival with minor or no improvement in fungal disease), progression of fungal disease, and death related to invasive fungal infection. The treatment response was undetermined in cases of death not related to invasive fungal infection, the absence of a radiological control at three months, or a duration of ISA treatment < 3 months. In addition, adverse events attributable to ISA were reported throughout treatment.

### 2.4. Statistical Analysis

Categorical variables are expressed as numbers (percentages) and continuous variables as medians [25th–75th percentiles]. Inter-individual variability was assessed by calculating the coefficient of variation (CV) of the mean ISA Cmin for all patients. Intra-individual variability could only be determined for patients who had at least three ISA Cmin determinations and is the mean of the CVs of all concentrations measured for a single patient.

A mixed-effect model was used to evaluate the influence of various factors on ISA Cmin. In the base mixed-effect model, random effects were included in the intercept for inter-individual variability. Univariate analyses were performed for categorical (gender and ISA route of administration) and continuous (age, weight, daily dose, treatment duration, ASAT, ALAT, GGT, ALP, total bilirubin, creatinine, CRP, protein, albumin, and LDH levels) variables using non-parametric tests (Mann-Whitney or Pearson tests). Covariates associated with a *p*-value < 0.2 in the univariate analysis were then included in the multivariate model. Two sets of analyses (univariate and multivariate) were performed; the first focused on ISA Cmin and the second on the Cmin of ISA weighted by the daily dose (ISA Cmin/D). The absence of collinearity was verified in the two multivariate models. The ALAT, ALP, GGT, and LDH levels, on the one hand, and the albumin levels, on the other, were therefore not included in the final model because they were collinear with the ASAT and protein levels, respectively. A *p*-value < 0.05 was considered statistically significant. All statistical analyses were performed using Jamovi^®^ (version 1.1.9) ([21]).

## 3. Results

### 3.1. Population Characteristics

**Figure 1:**
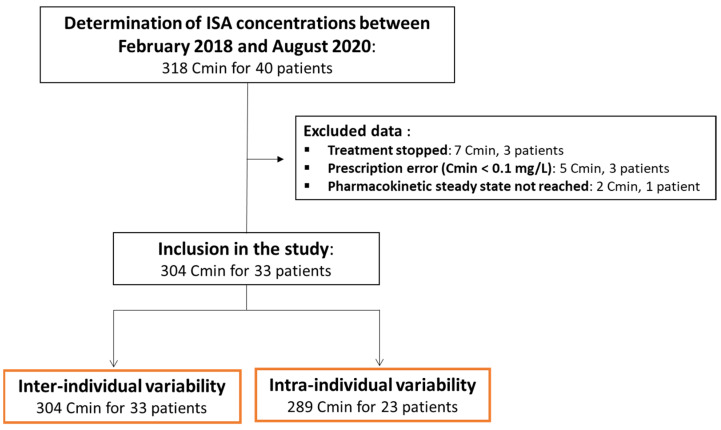
Flow chart.

The flow chart of the study is shown in Figure 1 and the baseline characteristics of the 33 included patients are presented in Table 1. Most patients (81.8%) had a hematological malignancy. ISA was indicated for the curative treatment of invasive aspergillosis for 29/33 (87.9%) patients (3 proven, 19 probable, and 7 possible invasive aspergillosis). It was indicated as first-line therapy for 14/33 (42.4%) patients.

### 3.2. Variability of ISA Concentrations

**Figure 2:**
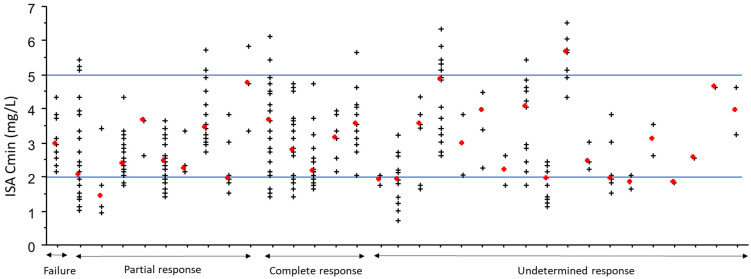
Variability of isavuconazole trough concentration (ISA Cmin) values for 33 patients according to the treatment response. Each vertical series of crosses corresponds to repeated ISA Cmin determinations for one patient, with the red cross indicating the median ISA Cmin per patient. Blue lines indicate the proposed therapeutic range at the Grenoble Alpes University Hospital.

In total, 304 ISA Cmin were analyzed (of which 91.1% determined during oral treatment), representing a median of 7 (1–6) ISA Cmin determinations per patient. The median ISA Cmin was 2.8 [2.0–3.7] mg/L. Inter- and intra-individual CVs were 41.5 and 30.7%, respectively (Figure 2). Sixty-six (22%) of 304 ISA Cmin for 16/33 (48%) patients were <2 mg/L and 25/304 (8%) for 9/33 (27%) patients were >5 mg/L. Certain cases for which the concentration fell outside those generally observed led to dose adjustments: 24 doses adjustments in 10 patients, for a median of 0 [0–1] dose adjustments per patient.

### 3.3. Determinants of the ISA Cmin

**Figure 3:**
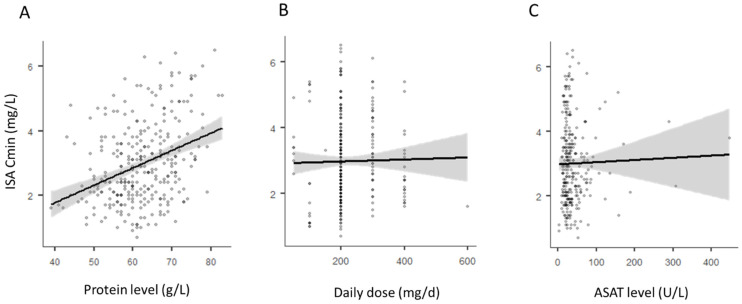
Link between isavuconazole trough concentrations (ISA Cmin) and protein level (**A**), daily dose (**B**) and ASAT level (**C**).

The results of univariate and multivariate analyses, considering ISA Cmin, and ISA Cmin/D, are presented in Table 2 and Table 3, respectively. In univariate analysis, Cmin and Cmin/D were positively associated with the levels of ASAT, ALAT, protein, and albumin, whereas they were not significantly associated with sex, age, route of administration, or the levels of ALP, CRP, creatinine, total bilirubin, or LDH. The duration of treatment was positively associated with the ISA Cmin only when weighted by the ISA dose (Table 3). Multivariate analyses showed that the dose, ASAT, and protein levels, were independently associated with the ISA Cmin (albumin level instead of protein level considering ISA Cmin/D). Figure 3 illustrates the link between ISA Cmin and the identified determinants.

### 3.4. Clinical Outcomes

Among the 25 patients with hematological malignancies curatively treated, the treatment response was evaluated for 15 (60.0%). The treatment response was undetermined in cases of death not related to invasive fungal infection (*n* = 1), the absence of a radiological control at three months in relation with palliative situation (*n* = 5), or a duration of ISA treatment < 3 months (*n* = 3). One additional patient could not be evaluated since ISA Cmin were measured too late in relation with timing of treatment response assessment. Among patients with determined treatment response, 5/15 (33.3%) patients showed a complete response and 9/15 (60.0%) a partial response. Treatment failure was observed for 1 patient with invasive aspergillosis associated with mucormycosis treated by ISA as third line (previous treatment by voriconazole and posaconazole), with minor improvement of clinical symptoms (pain especially) and radiological worsening (associated with repeated positive antigenemia). Patients with a complete or partial response had median ISA Cmin of 3.1 [2.8–3.5] and 2.4 [2.0–3.4] mg/L (see Figure 2). The unique patient with treatment failure received ISA as third line (after initial treatment by voriconazole and posaconazole). He benefited from 11 ISA Cmin measurements ranging from 2.9 to 4.3 mg/L with a median of 2.9.

Three of the 33 (9.1%) patients experienced adverse events related to ISA. One patient experienced vomiting, one a rash 24 h after the initiation of ISA, and one neutropenia. The median ISA Cmin for these patients were 3.9, 2.2, and 3.9 mg/L, respectively.

## 4. Discussion

This study, conducted on a large number of ISA Cmin (*n* = 304), showed that ISA exposure exhibits variability, with ISA dose, and ASAT and albumin levels, identified as independent factors associated with ISA Cmin.

The inter- and intra-individual CVs for ISA Cmin of 41.5 and 30.7% that we found shows that the ISA Cmin were variable, especially between patients. Such a finding is in agreement with those of several recent real-life studies that reported inter-individual CVs ranging from 36.6 to 61.5% [8,9,22,23]. Similarly, the intra-individual CV of 30.7% that we found is similar to the intra-individual CVs previously reported, ranging from 28.2 to 43.4% [9,10,22]. However, this level of variability is less than that observed for voriconazole [24] and posaconazole [25], other antifungal agents used to treat invasive fungal infections. These differences may be explained by the fact that ISA shows dose-proportional pharmacokinetics, with a long elimination half-life, leading to less variability in the concentration.

Variability of the ISA Cmin is independently related to the dose, as well as the levels of ASAT and albumin (or protein). The observed dose-dependent increase of the ISA Cmin is in accordance with the results of a previous study [10] and is logical, given its dose-proportional pharmacokinetics. The positive, independent association between ASAT levels and ISA Cmin has not been previously described. Indeed, the published phase III study did not show any association between ASAT or ALAT levels and ISA Cmin, but this observation was attributed to the fact that the ASAT and ALAT levels did not increase in the patients in their cohort [12]. More recently, a study performed in hematology patients reported an increase in the ISA Cmin associated with a decrease in GGT concentrations [9]. However, two studies showed that patients with liver dysfunction had reduced ISA clearance [26,27], which could lead to an increase in the ISA Cmin, as observed in our study. At the same time, hepatic dysfunction could also be a side effect of ISA, which complicates the interpretation of the association between ISA Cmin and ASAT levels. Univariate analyses showed the duration of treatment to not be associated with the ISA Cmin, whereas it was with increased ISA Cmin/Dose. This discrepancy between analyses is probably explained by the relatively frequent dose adjustments in our cohort (24 dose adjustments in 10 patients). The positive association between ISA Cmin/D and duration of treatment is in accordance with the results of several previous studies [9,10,23], one of which showed an increase of 0.032 mg/L per day of treatment [9]. Such accumulation of ISA over time is related to its very slow elimination and suggests the utility of ISA TDM in cases of prolonged treatment. Finally, the main factor that influences both the ISA Cmin and ISA Cmin/D is the albumin or protein level. Such a finding has not been previously reported but is logical, given the large fraction of ISA bound to albumin (>99%). Conversely, we did not find any association between ISA Cmin and sex [8] or age [10], contrary to previous studies, nor with inflammation. Indeed, CRP levels in our study were not related to the ISA Cmin, contrary to what has been observed for voriconazole [14,15,16,17]. This difference can be explained by the highly albumin bound of ISA and also its very long half-life, thus logically leading to a more stable Cmin. Indeed, even if an inflammatory episode occurred and could at least theoretically reduce the cytochrome P450 3A4-mediated metabolism of ISA [28], the expected increase in ISA exposure only affected the free fraction of ISA Cmin (<1%) and would only be visible after several days.

In our study, the treatment response could only be determined for approximately half of the included patients (*n* = 15), which did not allow to investigate the concentration-efficacy relationship. Similarly, the link between ISA Cmin and the toxic risk could not be assessed since adverse effects were largely underestimated (only three identified side effects) due to methodological considerations (retrospective design). This was, indeed, a limitation of our study. Several previous studies did not demonstrate any concentration-effect relationship, considering both efficacy [7] and toxicity [12,22]. Conversely, other studies have found an association between ISA concentrations and toxicity [9,10], suggesting an upper threshold of 5 mg/L to avoid the occurrence of gastrointestinal adverse effects [9]. Thus, these conflicting results let to think that further studies conducted on larger studies are needed to clarify the possible relationship between ISA exposure and its therapeutic and toxic effects.

Another limitation of this study, aside from its retrospective design and the limited number of patients, was that ISA exposure was evaluated by ISA Cmin only, considering the total form of ISA (both bound and unbound ISA). Indeed, the Cmin did not allow determination of pharmacokinetic parameters, such as clearance or the volume of distribution. In addition, as >99% of ISA is bound to albumin, the determination of unbound ISA Cmin would probably be more relevant, as recently shown for other anti-infective drugs highly bound to plasma proteins [28,29,30]. Finally, the inclusion of sample handled from the fourth day of treatment could lead to an overestimation of the variability of ISA Cmin. However, the high loading dose of ISA minimized this risk.

In conclusion, this study, conducted on a large number of ISA Cmin, shows that ISA exposure exhibits variability, with ISA dose, and ASAT and albumin levels identified to be independent factors associated with ISA Cmin. As >99% of ISA binds to plasma proteins, further studies are needed to investigate the variability of unbound ISA Cmin and a possible link between unbound ISA Cmin and drug efficacy and/or toxicity.

## Figures and Tables

**Table 1:** Patient characteristics.

Parameter	Value ^a^
Characteristic	
Demographics	
Age, years	60 [7–63]
Male	21 (63.6)
Weight, kg/m^2^	61.6 [53.8–77.3]
**Underlying disease**	
Hematological malignancy	27 (81.8)
Solid organ transplant	4 (12.1)
Others ^b^	2 (6.1)
**Biological variables**	
C-reactive protein (mg/L)	6.5 [4.0–28.3]
Aspartate aminotransferase (U/L)	29 [21–45]
Alanine aminotransferase (U/L)	45.5 [26–81]
Gamma glutamyltransferase (U/L)	264 [110–586]
Alkaline phosphatase (U/L)	143 [95.8–286]
Total bilirubin (µmol/L)	8.0 [5.0–11.0]
Creatinine (µmol/L)	78 [62–96]
Lactate dehydrogenase (U/L)	226 [182–279]
Protein (g/L)	62 [57–68]
Albumin (g/L)	35 [31–39]
**About ISA treatment**	
**ISA indication**	
Curative treatment	
Invasive aspergillosis	29 c (87.9)
Mucormycosis	4 c (9.1)
Prophylactic treatment	1 (3.0)
**Treatment line**	
First line	14 (42.4)
Second line	16 (48.5)
Third line	3 (9.1)
**Daily ISA maintenance dose (mg/day)**	200 [200–200]
**Route of administration**	
Oral	277 (91.1)
Intravenous	27 (8.9)
**Duration of treatment (days)**	95 [14–160]
**ISA Cmin (mg/L)**	2.8 [2.0–3.7]
**Number of ISA Cmin measurement per patient**	7 [2–16]
**Number of dose adjustment per patient**	0 [0–1]

ISA = isavuconazole. ^a^ Data are presented as numbers (%) or medians [25th–75th percentiles]. ^b^ Others include one case of cavum mass and one of ankylosing spondylitis. ^c^ One patient had mucormycosis associated with invasive aspergillosis.

**Table 2:** Factors that contribute to the variability of ISA Cmin.

Covariate	Available Data (%)	Univariate Analysis		Multivariate Analysis	
		Estimate ± SE	*p*-Value ^a^	Estimate ± SE	*p*-Value ^a^
Sex (Male/Female)	100	0.178 ± 0.110	**0.119**	0.297 ± 0.193	0.137
Age (years)	100	0.002 ± 0.004	0.607		
Weight (kg)	81.9	0.008 ± 0.003	**0.031**	−0.001 ± 0.005	0.781
Daily dose (mg/day)	100	0.002 ± 3.53 × 10^−4^	**<0.001**	0.004 ± 3.56 × 10^−4^	**<0.001**
Route of administration (oral/IV)	100	0.035 ± 0.097	0.721		
Treatment duration (days)	100	2.60 × 10^−4^ ± 3.32 × 10^−4^	0.434		
ASAT (U/L)	93.4	0.001 ± 5.21 × 10^−4^	**0.006**	0.002 ± 5.41 × 10^−4^	**0.002**
ALAT (U/L)	96.1	4.56 × 10^−4^ ± 2.08 × 10^−4^	**0.030**		
GGT (U/L)	96.1	−5.79 × 10^−6^ ± 5.08 × 10^−5^	0.909		
ALP (U/L)	96.1	−1.65 × 10^−5^ ± 1.69 × 10^−4^	0.922		
Total bilirubin (µmol/L)	96.1	−0.002 ± 0.002	0.426		
Creatinine (µmol/L)	98.7	1.80 × 10^−4^ ± 7.50 × 10^−4^	0.811		
CRP (mg/L)	93.4	−6.47 × 10^−4^ ± 5.64 × 10^−4^	0.252		
Protein (g/L)	98.4	0.014 ± 0.003	**<0.001**	0.022 ± 0.004	**<0.001**
Albumin (g/L)	73.4	0.012 ± 0.005	**0.011**		
LDH (U/L)	85.2	5.63 × 10^−4^ ± 3.60 × 10^−4^	0.120		

ASAT: aspartate aminotransferase, ALAT: alanine aminotransferase, GGT: gamma glutamyltransferase, ALP: alkaline phosphatase, CRP: C-reactive protein, LDH: lactate dehydrogenase, IV: intravenous. ^a^ Bold values indicate statistical significance.

**Table 3:** Factors that contribute to the variability of ISA Cmin after weighting by daily dose.

Covariate	Available Data (%)	Univariate Analysis		Multivariate Analysis	
		Estimate ± SE	*p*-Value ^a^	Estimate ± SE	*p*-Value ^a^
Sex (Male/Female)	100	0.246 ± 0.154	0.120	0.321 ± 0.228	0.176
Age (years)	100	0.007 ± 0.006	0.238		
Weight (kg)	81.9	−0.001 ± 0.005	0.764		
Route of administration (oral/IV)	100	0.045 ± 0.103	0.665		
Treatment duration (days)	100	0.001 ± 3.31 × 10^−4^	**<0.001**	7.29 × 10^−4^ ± 4.35 × 10^−4^	0.095
ASAT (U/L)	93.4	0.001 ± 5.07 × 10^−4^	**<0.001**	0.002 ± 5.97 × 10^−4^	**0.001**
ALAT (U/L)	96.1	6.38 × 10^−4^ ± 2.03 × 10^−4^	**0.002**		
GGT (U/L)	96.1	1.23 × 10^−4^ ± 5.22 × 10^−5^	**0.019**		
ALP (U/L)	96.1	2.99 × 10^−4^ ± 1.76 × 10^−4^	0.092		
Total bilirubin (µmol/L)	96.1	−0.004 ± 0.003	0.181		
Creatinine (µmol/L)	98.7	−0.001 ± 7.91 × 10^−4^	0.187		
CRP (mg/L)	93.4	−0.001 ± 5.54 × 10^−4^	0.070	2.84 × 10^−4^ ± 7.06 × 10^−4^	0.688
Protein (g/L)	98.4	0.018 ± 0.004	**<0.001**		
Albumin (g/L)	73.4	0.027 ± 0.004	**<0.001**	0.025 ± 0.006	**<0.001**
LDH (U/L)	85.2	1.26 × 10^−4^ ± 3.58 × 10^−4^	0.726		

ASAT: aspartate aminotransferase, ALAT: alanine aminotransferase, GGT: gamma glutamyltransferase, ALP: alkaline phosphatase, CRP: C-reactive protein, LDH: lactate dehydrogenase, IV: intravenous. ^a^ Bold values indicate statistical significance.

## Data Availability

The data that support the findings of this study are available from the corresponding author upon reasonable request.

## Abbreviations

ALAT	alanine aminotransferase
ALP	alkaline phosphatase
ASAT	aspartate aminotransferase
Cmin	trough concentrations
CRP	C-reactive protein
CV	coefficient of variation
CYP	cytochrome P450
GGT	gamma glutamyltransferase
ISA	isavuconazole
LDH	lactate dehydrogenase
TDM	Therapeutic drug monitoring
